# American Dental Convention

**Published:** 1863-07

**Authors:** W. B. Roberts


### AMERICAN DENTAL CONVENTION.

The ninth Annual Session of this body will take place on Tuesday, August 4th, at Saratoga Springs, New York. The members of the profession from all parts of the country are cordially invited to be present. The Executive Committee, appointed by the President to prepare the Order of Business for 1863, reported the following subjects for discussion, which were adopted:

I. Causes Influencing an Abnormal Development of the Teeth.

II. Treatment of Dental Irregularities, and Appliances for the same.

III. 1. Filling Teeth. 2. Filling Temporary Teeth. 3. Best Material for same.

IV. Diseases of the Antrum, and Treatment.

V. Treatment of Cleft Palate.

VI. Alveolar Abscess.

VII. Mechanical Dentistry.

VIII. Miscellaneous Business.

W. H. DWINELLE,<br>

W. A. PEASE,<br>

W. D. STONE,<br>

D. W. PERKINS,<br>

T. L. BUCKINGHAM, } *Committee.*

Although this is technically termed the *American* Dental Convention, dentists from all parts of the world are always welcome, and invited to take part in the discussions.

W. B. ROBERTS, *Cor. Secretary.*

---

For the Register.

FAST FILLINGS.—Mr. Allport, of Chicago, is well known to the profession as a tip top Dentist.

Yet he is a fast man, not in the Young America sense, but as an operator.

We have seen him fill teeth with great rapidity during the past week, in his own office, and it has occurred to us that an article on this subject from his ready pen might benefit the profession. We wait a response. G. F. F.

